# Association of birth weight, childhood obesity, and age at menarche with the risk of ovarian dysfunction: A mendelian randomization study

**DOI:** 10.1371/journal.pone.0306365

**Published:** 2024-07-18

**Authors:** Chunxiao Dang, Jianjuan Li, Xiao Yu, Jinxing Liu, Pengfei Liu, Xiaoling Yang

**Affiliations:** 1 First Clinical Medical College, Shandong University of Traditional Chinese Medicine, Jinan, Shandong, China; 2 Dongying People’s Hospital (Dongying Hospital of Shandong Provincial Hospital Group), Dongying, Shandong, China; 3 Department of Gynaecology, Affiliated Hospital of Shandong University of Traditional Chinese Medicine, Jinan, Shandong, China; 4 Department of Pediatrics, Affiliated Hospital of Shandong University of Traditional Chinese Medicine, Jinan, Shandong, China; National Institute of Child Health and Human Development (NICHD), NIH, UNITED STATES OF AMERICA

## Abstract

**Background:**

Observational studies have revealed associations between birth weight, childhood obesity, age at menarche, and ovarian dysfunction. However, these studies are susceptible to unavoidable confounding factors, leading to ongoing debates regarding their conclusions and making causal relationships challenging to infer. In light of these challenges, Mendelian randomization was employed in this study to investigate the causal relationships between birth weight, childhood obesity, age at menarche, and ovarian dysfunction.

**Methods:**

This study employed a two-sample Mendelian randomization approach using genetic variation as instrumental variables to investigate causal relationships. Genetic variation data were sourced from summary data of genome-wide association studies in European populations. Instrumental variables were selected based on the principles of Mendel’s three assumptions. The study utilized the inverse variance weighted method to assess the relationships between birth weight, childhood obesity, age at menarche, and ovarian dysfunction. Supplementary analyses were conducted using MR-Egger regression, the weighted median method, and the weighted median mode to complement the IVW results. Furthermore, the study conducted heterogeneity, horizontal pleiotropy, and sensitivity analyses to evaluate the robustness of the results.

**Results:**

Based on the inverse variance weighted method, it was found that there exists a causal relationship between childhood obesity (OR = 1.378, 95% CI: 1.113∼1.705, p = 0.003), age at menarche (OR = 0.639, 95% CI: 0.468∼0.871, p = 0.005), and ovarian dysfunction, while no causal relationship was observed between birth weight and ovarian dysfunction. Heterogeneity tests, multiplicity tests, and leave-one-out sensitivity analyses did not detect any heterogeneity or multiplicity effects in the estimated impact of these three exposure factors on the risk of ovarian dysfunction.

**Conclusions:**

This study represents the first evidence suggesting a potential causal relationship between childhood obesity, age at menarche, and ovarian dysfunction. Childhood obesity was found to increase the risk of ovarian dysfunction, while a later age at menarche was associated with a reduced risk of ovarian dysfunction.

## Introduction

Ovarian reserve refers to a woman’s capacity for the growth, development, and formation of potentially fertilizable oocyte cells within the ovaries. It encompasses both the quantity of eggs produced within a woman’s ovaries and the potential quality of those eggs. Ovarian dysfunction, on the other hand, refers to a condition where the number of eggs retained within a woman’s ovaries does not meet the threshold criteria, leading to a decline in her reproductive capacity [1]. Ovarian reserve and ovarian aging have become prominent areas of focus in current clinical practice and research.

There is a hypothesis suggesting that birth weight is influenced by genetic factors from both parents as well as the intrauterine environment [2]. Further research has indicated that girls born with small gestational age (SGA) tend to have a reduced ovulation rate during pregnancy [3], but limited sample sizes may introduce bias into these findings. Childhood obesity is a significant public health concern, with reported rates as high as 12.7% in the United States [4]. Previous clinical studies and meta-analyses have shown that adult obesity has a negative impact on ovarian function [5, 6]. However, there has been limited research investigating whether childhood obesity similarly affects ovarian function. Age at menarche can serve as a reflection of a female’s hormonal levels during puberty and is influenced by genetic, epigenetic, and environmental factors [7]. Some studies have suggested that age at menarche is not correlated with ovarian function [8], but the limited sample sizes and potential methodological flaws in traditional observational studies raise questions about these findings. Therefore, further research is needed to clarify the relationships between birth weight, childhood obesity, age at menarche, and ovarian dysfunction.

Mendelian randomization (MR) employs single nucleotide polymorphisms (SNPs) as instrumental variables (IVs) to infer causal relationships between exposures and outcomes. By adhering to the genetic principle of "random allocation of parental alleles to offspring", it achieves a quasi-randomized grouping effect similar to that of a randomized controlled trial, while mitigating the influence of external environmental factors. This allows MR to address the limitations of observational studies [9]. In this study, a two-sample Mendelian randomization approach was employed. Leveraging publicly available large-scale Genome-Wide Association Study (GWAS) databases, it aimed to investigate the causal relationships between birth weight, childhood obesity, age at menarche, and ovarian dysfunction.

## Material and methods

### Research design

**Fig 1:**
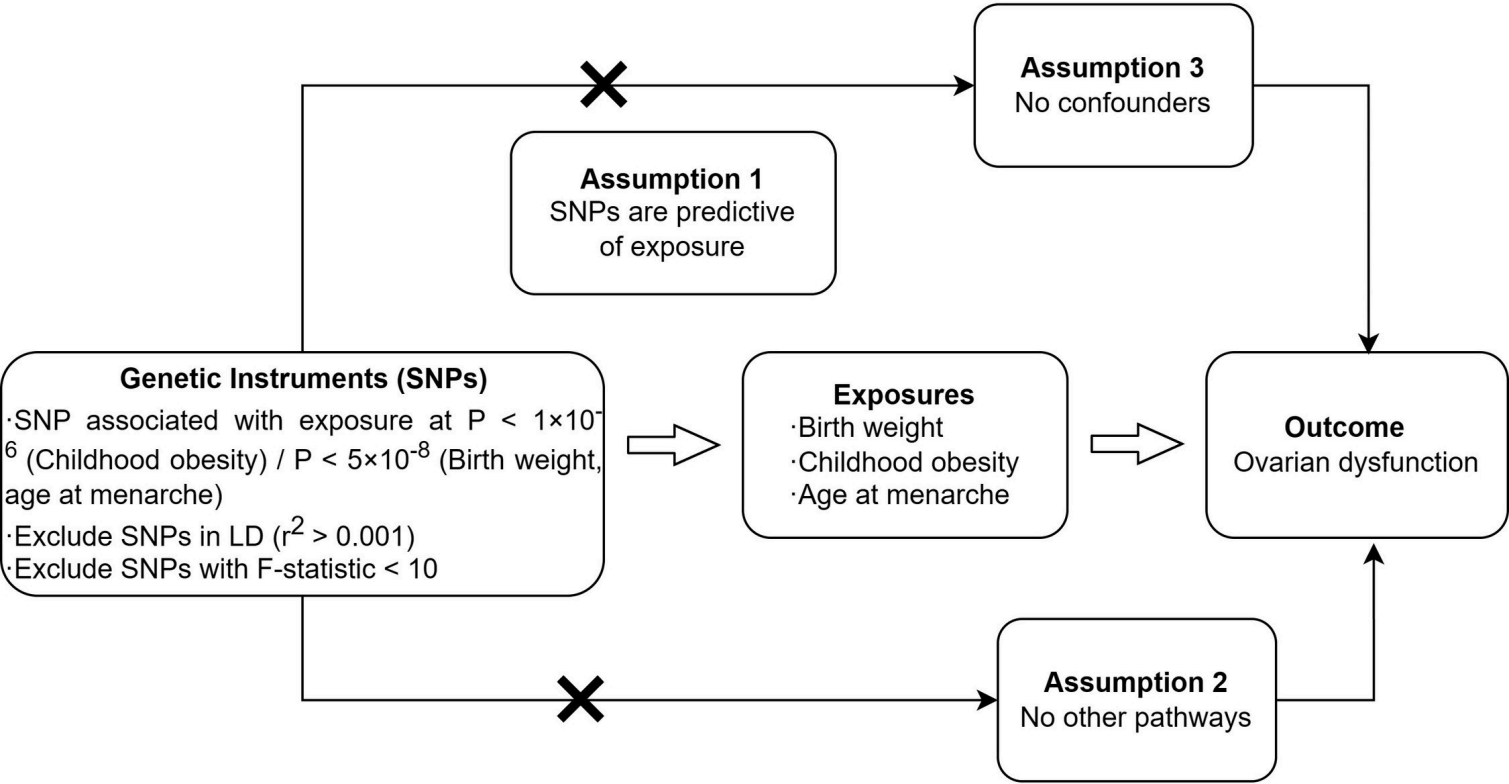
Overview of MR rationale, design, and procedures.

In determining where the GWAS summary data for the exposure variable and the outcome variable are assumed to be mutually independent, we conducted association analyses using the TwoSampleMR package in the R programming language. Specifically, we utilized birth weight, childhood obesity, and age at menarche as exposure variables, with ovarian dysfunction serving as the outcome variable. The objective was to investigate whether a causal effect exists between these exposure variables and the outcome variable, with the specific design outlined in Fig 1. MR analysis adheres to the following three key assumptions [10]:

1. Instrumental variables have a strong correlation with the exposure variables (birth weight, childhood obesity, age at menarche).
2. Instrumental variables are independent of both observed and unobserved confounding factors.
3. Instrumental variables solely influence the outcome (ovarian dysfunction) through the exposure variables.

### Data source

The summary data for birth weight were sourced from the IEU OpenGWAS project database (https://gwas.mrcieu.ac.uk/), encompassing 143,677 participants. Data for childhood obesity came from the Early Growth Genetics (EGG) Consortium (http://egg-consortium.org/), comprising 13,848 participants (5,530 cases and 8,318 controls) [11]. Age at menarche summary data was obtained from the ReproGen Consortium (https://reprogen.org/), involving 182,416 participants [12]. Ovarian dysfunction summary data were acquired from the Finngen database, with a total of 16,379,685 SNPs in 119,170 individuals (https://gwas.mrcieu.ac.uk/datasets/finn-b-E4_OVARDYS/). The exposure and outcome summary data were derived from two separate databases to prevent potential data sample overlap. The samples included both males and females and were sourced from European populations. As our study is based on publicly available databases, ethical committee approval was not required.

### Instrumental variable selection

1. IVs Selection: To obtain strongly associated exposure data, birth weight and age at menarche were screened with a significance threshold of P < 5 × 10^-8 [13]. However, under this genome-wide significance threshold, most of the childhood obesity-related SNPs failed to be identified. Therefore, for childhood obesity, a more lenient P-value threshold of P < 1 × 10^-6 was set to select associated SNPs. (2) Independence Criterion: The linkage disequilibrium (LD) among SNPs for each risk factor was assessed using PLINK clustering. SNPs with LD coefficient r^2 greater than 0.001 and located within a physical distance of less than 10,000 kb were excluded. This step ensured the mutual independence of SNPs to eliminate the influence of genetic pleiotropy on outcomes [14, 15]. (3) Statistical Strength Criteria: The effectiveness of instrumental variables was assessed using the F-statistic, calculated as F = β^2/SE^2 (where β represents the effect size of the allele and SE is the standard error). Instrumental variables with F-values less than 10 were excluded, ensuring that the instrumental variables were unrelated to unmeasured confounding factors [16]. Finally, the "harmonise_data" function from the Two Sample MR package was employed to align the directions of effect alleles for exposure and outcome variables, remove palindromic and incompatible SNPs, and consolidate P-values while excluding SNPs highly correlated with ovarian dysfunction (P < 5 × 10^-8) [17].

### Mendelian randomization analysis

In this study, the inverse variance weighted (IVW) method [18] was employed as the primary analytical approach for establishing causal relationships. This method, assuming the validity of all instrumental variables, calculates weighted estimates by taking the reciprocal of their variances as weights. It provides the most accurate results when there is no heterogeneity or horizontal pleiotropy present. Additionally, MR-Egger regression, the weighted median (WME) method, and weighted mode (WM) were used as supplementary analyses to complement the IVW results.

### Sensitivity analysis

Heterogeneity testing [19] assesses the presence of differences among various IVs. It utilizes the P-value from Cochran’s Q test to evaluate heterogeneity, with P > 0.05 indicating the absence of heterogeneity. If heterogeneity is detected, the MR pleiotropy residual sum and outlier (MR-PRESSO) test is employed to assess potential outliers [20], eliminate them, and then reanalyze the data. Multiplicity testing [21] verifies the reliability of MR analysis results. MR-Egger intercept is used to detect horizontal pleiotropy, with P > 0.05 indicating the absence of horizontal pleiotropy and, thus, the reliability of the MR analysis results. Sensitivity testing [22] is conducted using a "leave-one-out" approach, sequentially removing each SNP. If the MR results derived from the remaining SNPs do not exhibit significant differences from the overall result, it demonstrates the robustness of the MR results.

## Results

### Validity of instrumental variables

Following the selection process, the study obtained 2,264, 365, and 2,454 SNPs strongly correlated with birth weight, childhood obesity, and age at menarche, respectively. After pruning for linkage disequilibrium, these numbers were reduced to 50, 8, and 68 SNPs, respectively. Subsequently, palindrome and incompatible SNPs were identified and removed. Additionally, a search was conducted in the PhenoScanner database to identify any SNPs associated with potential confounding factors. In the final selection, a total of 42 SNPs for birth weight, 7 SNPs for childhood obesity, and 54 SNPs for age at menarche were included as instrumental variables. Moreover, all SNPs had F-statistics exceeding 10, indicating the effectiveness of the selected instrumental variables. Weak instrument bias was not expected to affect the causal inference results in this MR analysis. Basic information for some of the instrumental variables is provided in Table 1, while comprehensive details for all instrumental variables can be found in S1 Data.

**Table 1:** The top 5 SNPs with the strongest correlation with birth weight, childhood obesity, and age at menarche.

Exposure	SNP	Effect_allele	β	S.E.	Pval.outcome	Pval.exposure
Birth weight	rs900399	G	-0.052	0.004	0.315	2.200E-41
	rs1351394	C	-0.044	0.004	0.971	1.900E-32
	rs17034876	T	0.047	0.004	0.969	2.600E-29
	rs35261542	A	-0.044	0.004	0.510	4.400E-27
	rs11720108	T	0.046	0.004	0.352	3.400E-26
Childhood obesity	rs9941349	T	0.198	0.027	0.773	1.160E-13
	rs4854344	T	0.245	0.035	0.018	3.220E-12
	rs6752378	A	0.170	0.026	0.761	1.050E-10
	rs571312	A	0.199	0.031	0.125	1.250E-10
	rs7138803	A	0.167	0.027	0.194	6.500E-10
Age at menarche	rs1516883	A	-0.091	0.003	0.058	1.000E-200
	rs2153127	C	-0.077	0.002	0.769	1.000E-200
	rs11715566	T	0.052	0.006	0.038	2.600E-20
	rs9635759	A	0.058	0.006	0.984	7.700E-20
	rs466639	C	0.075	0.009	0.140	7.000E-18

### Association of birth weight, childhood obesity, and age at menarche with the overall risk of PCOS

**Fig 2:**
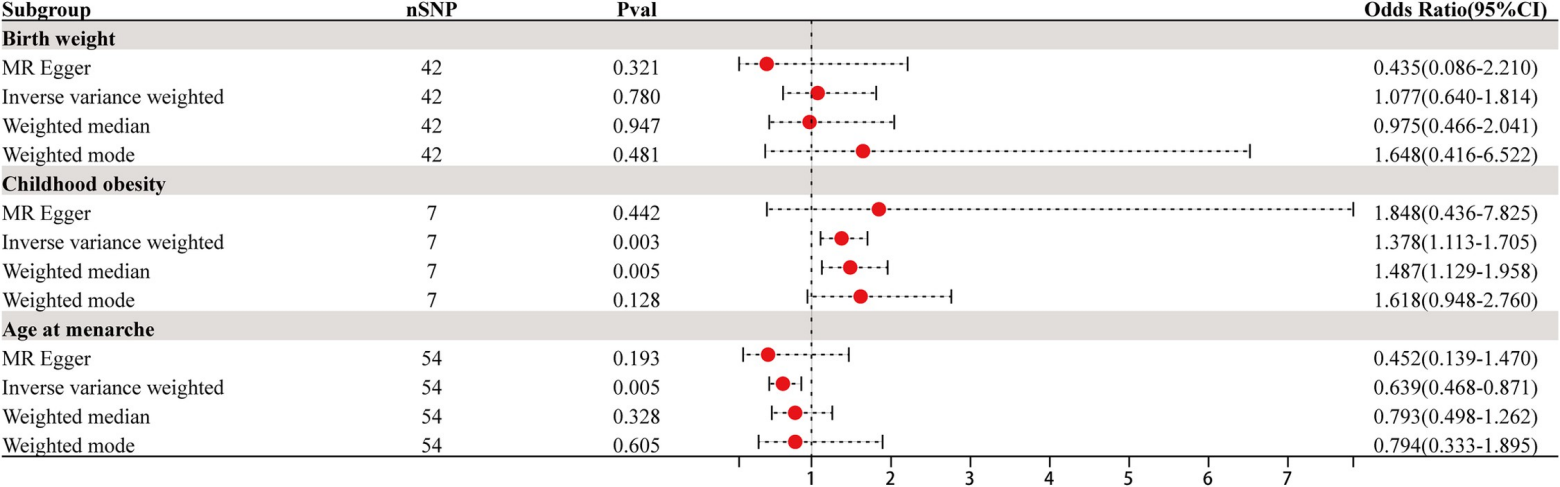
Forrest plot for causal associations of birth weight, childhood obesity, and age at menarche with ovarian dysfunction risk based on four MR methods.

**Fig 3:**
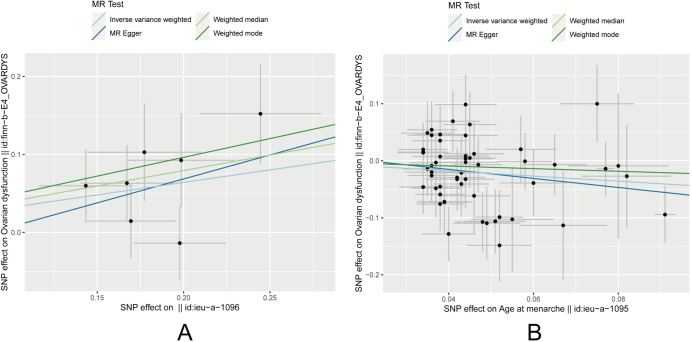
Scatter plot for causal associations of (A) childhood obesity and (B) age at menarche with ovarian dysfunction risk based on four MR methods.

As both heterogeneity and pleiotropy tests yielded negative results, the IVW analysis results were considered the primary reference in this study. The MR analysis results indicated statistical significance with P-values of 0.003 for childhood obesity and 0.005 for age at menarche. These results suggest a causal relationship between childhood obesity, age at menarche, and the occurrence of ovarian dysfunction. Furthermore, childhood obesity showed a positive association with ovarian dysfunction (OR = 1.378, 95% CI: 1.113∼1.705), while age at menarche exhibited a negative association (OR = 0.639, 95% CI: 0.468∼0.871). These findings are illustrated in Figs 2 and 3. Additionally, there was no causal relationship between birth weight and ovarian dysfunction, with a P-value of 0.780, indicating a lack of statistical significance (P > 0.05).

### Sensitivity analysis of MR analysis

**Fig 4:**
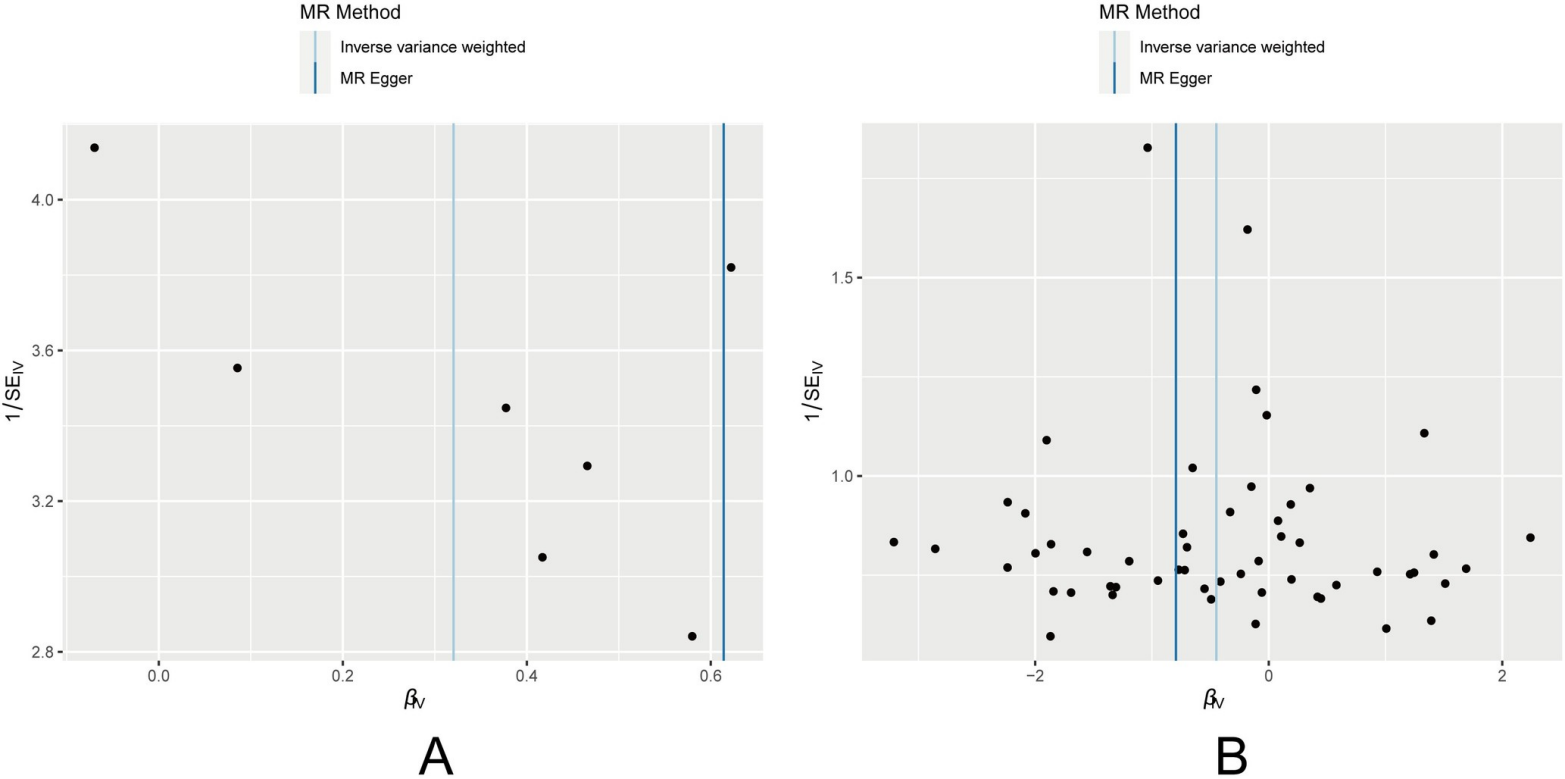
Funnel plot of the MR analysis of (A) childhood obesity and (B) age at menarche on ovarian dysfunction.

**Fig 5:**
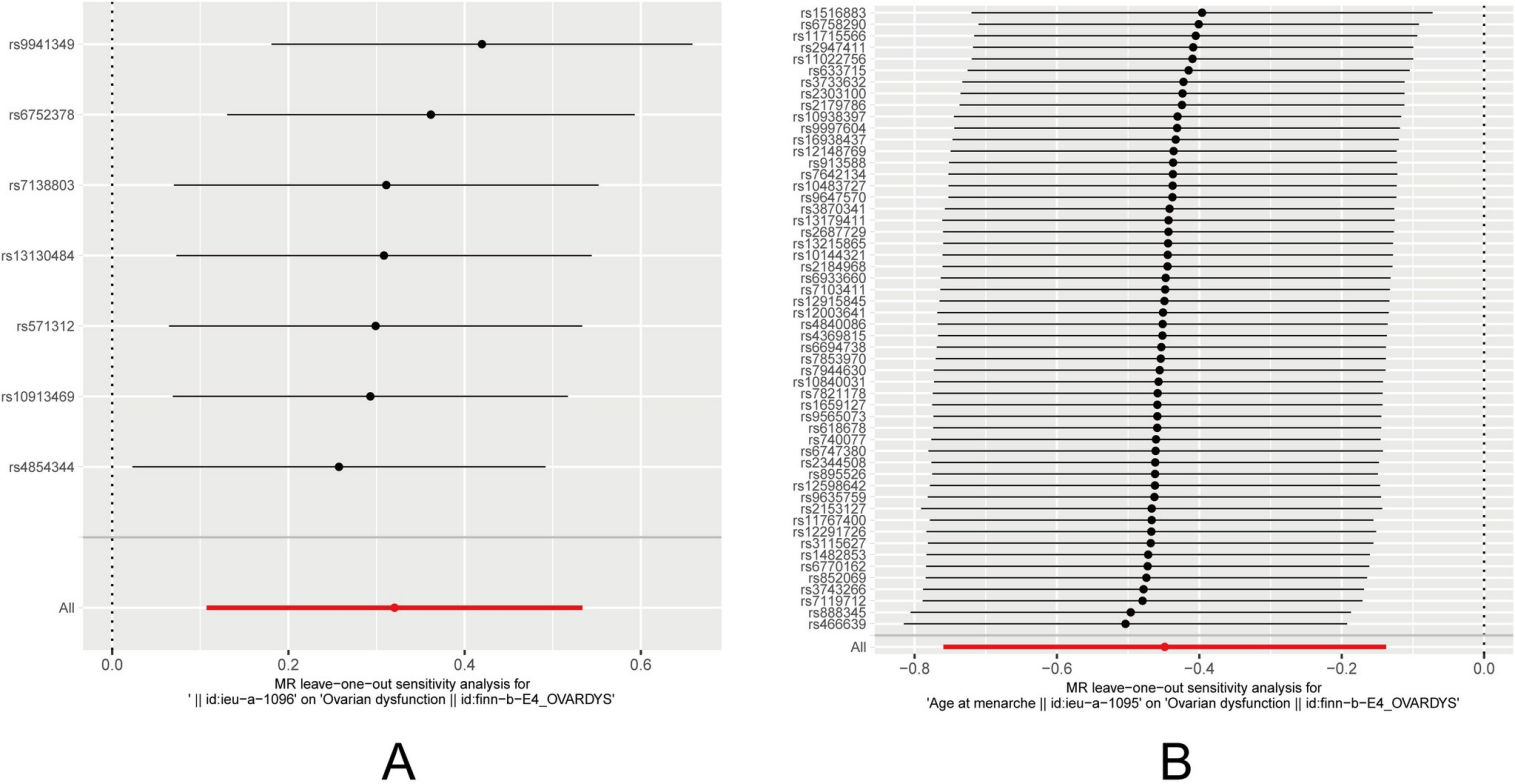
Leave-one-out regression analysis of (A) childhood obesity and (B) age at menarche on ovarian dysfunction.

An examination of the heterogeneity revealed that the funnel plot for childhood obesity did not exhibit the typical symmetrical shape (Fig 4). To eliminate subjective factors, further analysis computed Cochran’s Q-values with a distribution set at 10,000. Cochran’s Q tests indicated the absence of heterogeneity among SNPs. Moreover, the results from the pleiotropy test showed that the MR-Egger regression intercept p-values were all greater than 0.05, indicating the absence of horizontal pleiotropy (Table 2). In the sensitivity analysis using the "leave-one-out" approach, the removal of any individual SNP did not significantly alter the IVW analysis results, and the remaining SNPs yielded results similar to those of the entire dataset (Fig 5). Additionally, the MR-PRESSO analysis did not detect any significantly influential SNPs, indicating the absence of SNPs with a substantial impact on causal associations.

**Table 2:** Heterogeneity and pleiotropy evaluations for genetically causal associations of birth weight, childhood obesity, and age at menarche with ovarian dysfunction risk.

Exposure	Cochran’s Q		MR-Egger	
	Q	Q_pval	egger_intercept	pval
Birth weight	35.357	0.974	0.031	0.255
Childhood obesity	5.530	0.478	-0.054	0.703
Age at menarche	54.493	0.417	0.017	0.553

## Discussion

### Main findings and interpretation

This study employed a two-sample Mendelian randomization approach using GWAS data to investigate the causal relationships between birth weight, childhood obesity, age at menarche, and ovarian dysfunction. The results indicate that childhood obesity increases the risk of ovarian dysfunction in women (OR = 1.378, 95% CI: 1.113∼1.705), and an earlier age at menarche is associated with a greater risk of ovarian dysfunction (OR = 0.639, 95% CI: 0.468∼0.871). However, there is no causal relationship between birth weight and ovarian dysfunction.

Due to the original ovarian follicle pool being established within the uterus, it may be influenced by parental characteristics and the intrauterine environment [23]. Additionally, a study in 2007 [24] suggested that newborns with relatively lower birth weights have smaller ovarian volumes, raising the hypothesis that smaller ovarian volumes and ovarian dysfunction in females may originate during fetal development. However, a study in 2011 found no significant differences in follicular-phase LH, FSH, E_2_, AMH levels, or the response to endogenous GnRH between small for gestational age and appropriate for gestational age females [25]. This suggests that there may be no correlation between female ovarian function and birth weight. The contrasting results between these two studies could be attributed to the challenges of conducting extensive clinical follow-ups, resulting in limited sample sizes and potential biases influenced by various confounding factors.

A study conducted in 2014, which included 103 participants, found a higher prevalence of ovarian dysfunction among females who had insufficient weight during childhood [26]. Recent research has consistently indicated that childhood obesity leads to an earlier onset of puberty in females, increases the risk of developing polycystic ovary syndrome in adulthood, and affects fertility, among other factors [27, 28]. However, studies exploring the relationship between childhood obesity and adult ovarian function have been lacking. In contrast to previous research, our study discovered that females who were obese during childhood had a higher risk of developing ovarian dysfunction. This could potentially be attributed to the coordinated actions of various hormones such as leptin, insulin, and adrenaline [29]. The SNP rs9941349, located within the FTO gene intron, has been associated with abnormalities in FTO and linked to impaired oocyte maturation and premature ovarian failure. However, the precise mechanisms involved still require further elucidation through future research [30].

The earliest study from 2012 [31] found that women who experienced an earlier age at menarche had higher AMH levels during their youth, but it did not conduct longer-term research. In contrast, a study by Andrea Weghofer and colleagues in 2013 [32] suggested that an earlier age at menarche was associated with a higher risk of ovarian dysfunction. This association may be related to factors like follicular pool size and the speed of follicle recruitment. However, studies from 2018 and 2021 [8, 33] did not find any significant relationship between age at menarche and female ovarian function. The question of whether age at menarche is related to or causally linked to ovarian function in women is complex and may be influenced by various factors such as lifestyle and reproductive factors, making it challenging to provide a definitive answer through observational studies alone.

In contrast to previous observational studies, which can be susceptible to various confounding factors like genetics and immunity, and considering the limitations of conducting large-scale RCT clinical trials, this MR analysis study utilized openly accessible GWAS data. The results are transparent and publicly available. The selected data populations were exclusively of European descent, minimizing biases stemming from racial factors. This approach also aimed to reduce the inherent biases in observational studies that may arise due to residual confounding or reverse causality. Furthermore, it leverages a causal temporal framework [34]. To ensure the credibility of the study, heterogeneity tests were conducted. The P-values for both IVW and MR-Egger were greater than 0.05, indicating the absence of heterogeneity. Multiple-effect tests yielded P-values greater than 0.05, indicating that the results were not affected by multiple effects. Sensitivity analysis using the "leave-one-out" method also confirmed the stability and reliability of the findings. Our data support a causal relationship between childhood obesity, age at menarche, and ovarian dysfunction while not supporting a causal relationship between birth weight and ovarian dysfunction.

### Limitation

However, our study has several limitations: (1) MR assumes a linear relationship between exposure and outcome. Therefore, it may not be applicable if the relationship between the two is nonlinear. (2) MR analysis can only explore causal relationships and cannot investigate specific biological mechanisms. (3) The outcome data used in the study come from European populations, and whether the results are representative of the entire population remains to be verified. (4) The data obtained lack more detailed cohort information such as age and gender, which limits the possibility of conducting subgroup analyses.

## Conclusions

In summary, this study employed Mendelian randomization to analyze the causal relationships between birth weight, childhood obesity, age at menarche, and ovarian dysfunction. The results indicate that childhood obesity increases the risk of ovarian dysfunction, while a later age at menarche is associated with a reduced risk. This finding further confirms the positive correlation between age at menarche and ovarian function. Additionally, maintaining a healthy BMI during childhood is equally crucial for preserving ovarian function. Nevertheless, larger experimental studies are needed to elucidate the specific mechanisms through which childhood obesity and age at menarche impact ovarian function since ovarian dysfunction remains a disease with unclear etiology.

## Supporting information

S1 Data All SNP information of birth weight, childhood obesity and age at menarche. (XLSX)
