# Correction: Association of birth weight, childhood obesity, and age at menarche with the risk of ovarian dysfunction: A mendelian randomization study

**DOI:** 10.1371/journal.pone.0314370

**Published:** 2024-11-20

**Authors:** Chunxiao Dang, Jianjuan Li, Xiao Yu, Jinxing Liu, Pengfei Liu, Xiaoling Yang

An affiliation is missing for the first author. Chunxiao Dang is also affiliated with #3: Department of Gynaecology, Affiliated Hospital of Shandong University of Traditional Chinese Medicine, Jinan, Shandong, China.
